# Neuropathic Pain Medication and Antidepressant Use after Disability Pension in Patients with Spinal Cord Stimulation for Persistent Spinal Pain Syndrome

**DOI:** 10.1155/2024/4953758

**Published:** 2024-01-31

**Authors:** Hanna Kaijankoski, Mette Nissen, Tiina-Mari Ikäheimo, Mikael von und zu Fraunberg, Olavi Airaksinen, Jukka Huttunen

**Affiliations:** ^1^Departments of Physical and Rehabilitation Medicine, Kuopio University Hospital, PB 100, KYS 70029, Kuopio, Finland; ^2^University of Eastern Finland, Kuopio, Finland; ^3^Neurosurgery, Kuopio University Hospital, PB 100, 70029 KYS, Kuopio, Finland; ^4^Neurosurgery, Oulu University Hospital, Kajaanintie 50, Oulu 90220, Finland; ^5^University of Oulu, Oulu, Finland

## Abstract

**Background:**

Treatment of persistent spinal pain syndrome (PSPS) is challenging. Chronic pain associated with PSPS can lead to an impaired ability to work.

**Objective:**

To obtain information on whether receiving a disability pension (DP) affects pain and pain treatments in retiring working-age PSPS patients. Neuropathic pain medication and antidepressant use were considered as an indicator of neuropathic pain.

**Methods:**

The study group comprised 129 consecutive PSPS patients with spinal cord stimulation (SCS) devices implanted at Kuopio University Hospital Neurosurgery between January 1, 1996, and December 31, 2014. Purchase data of gabapentinoids, tricyclic antidepressants, and serotonin-norepinephrine reuptake inhibitors from January 1995 to March 2016, as well as the data on working ability, were retrieved from national registries.

**Results:**

The data showed that 28 of 129 (21.7%) SCS permanent patients had a DP, and 27 had a sufficient follow-up time (two years before and one year after DP). Most patients (61%) used neuropathic pain medications during the follow-up, while 44% used antidepressants. Most patients (70%, *n* = 19) retired because of dorsopathies. The dose of gabapentinoids started to increase before the DP; after the DP, the doses started to increase again after the decrease but remained at a lower level.

**Conclusions:**

Neuropathic pain medication and antidepressant use suggest that pain continues after the DP—that is, pensioners continue to experience inconvenient chronic pain. Resources for patient care are therefore needed after the DP. However, the DP reduces the dose increase of gabapentinoids; the dose is higher immediately before retirement than at the end of the follow-up.

## 1. Introduction

A significant portion of patients who have undergone lumbar spinal surgery continue to suffer from persistent postoperative pain. This condition has been traditionally labeled as “failed back surgery syndrome” (FBSS), but the ICD-11 now recommends the more appropriate and less misleading term “persistent spinal pain syndrome” (PSPS) [1, 2]. PSPS patients usually suffer from chronic back pain and/or pain in the lower extremities, which can lead to increased disability, decreased quality of life, and impaired ability to work [3, 4].

The first-line therapy for PSPS is to optimize nonmedical and medical treatment [5]. The pharmacological treatment of PSPS with a neuropathic radicular component involves the use of gabapentinoids, tricyclic antidepressants (TCA), and serotonin-norepinephrine reuptake inhibitors (SNRIs: duloxetine and venlafaxine) [5, 6]. Spinal cord stimulation (SCS) has been found to be effective in reducing neuropathic leg pain [7, 8].

The purpose of this study was (1) to investigate the prevalence and dosing of gabapentinoids, TCA, and SNRI in retiring PSPS patients, (2) to obtain indirect information on whether receiving a disability pension (DP) affects pain and pain treatments, and (3) to communicate information to clinicians about patients' need for pain treatments after receiving a DP.

## 2. Materials and Methods

Between January 1, 1996, and December 31, 2014, 230 consecutive PSPS patients underwent an SCS trial with surgical paddle lead at Kuopio University Hospital (KUH) Neurosurgery. KUH is a publicly funded nonprofit hospital in Eastern Finland.

In this article, we refer consistently to PSPS type 2 patients. PSPS type 2 was defined as radicular lower limb pain or combined lumbar pain after one or multiple lumbar surgeries due to spinal stenosis or disk herniation. PSPS diagnosis was made by a neurosurgeon, orthopedic surgeon, or pain physician. Patients were offered conservative treatment, such as oral analgesics and physical therapy. Following a policlinic evaluation, the decision to try an SCS device (trial period) was made by a neurosurgeon together with the patient.

The SCS paddle-lead electrode (Resume 3586, Specify 2 × 4 3998, or Specify 5-6-5 39565, Symmix 3982, Medtronic, Minneapolis, MN, USA) was implanted into the epidural space microsurgically. After a trial period (range 2–15 days), a permanent internal pulse generator (IPG; model 7425, model 37703, model 7427V, model 37702, or model 97702, Medtronic) was fitted in 179 of 230 (78%) patients who experienced significant pain relief following SCS paddle-lead electrode implantation. The electrode was removed from patients who did not experience adequate pain relief during the trial period (*n* = 51).

**Figure 1:**
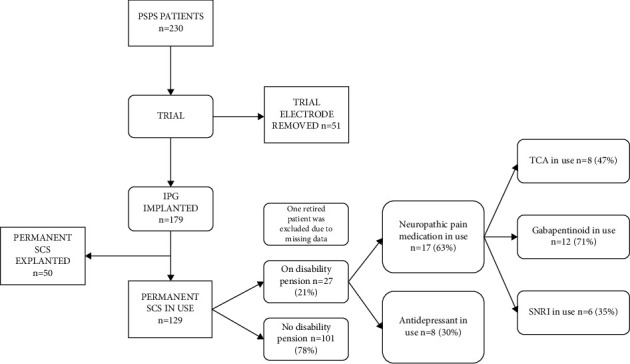
Disability pensions of 129 persistent spinal pain syndrome patients with spinal cord stimulation in use at the end of follow-up and their neuropathic pain medication and antidepressant usage during the follow-up (2 y before and 1 y after disability pension) based on data from the Social Insurance Institute of Finland.

Of the 179 patients, 129 (72%) used SCS at the end of the follow-up, and 50 (28%) had their SCS hardware explanted. Of the 129 patients (study group), 28 (22%) retired during the study period, and one was excluded due to missing data. Patients who underwent a trial only or had their SCS hardware explanted were excluded from this study (Figure 1). Patients' medical records and added data from national registers were retrospectively assessed.

The Finnish social security and pension system has previously been described [9]. Medication purchase data were retrospectively retrieved from a nationwide registry. In Finland, all prescription medications, prescription dates, purchase dates, amounts, and prices are stored in a registry maintained by the Social Insurance Institution (SII). We obtained information on all purchases of prescribed gabapentinoids, TCAs, opioids, serotonin-norepinephrine reuptake inhibitors (SNRIs), and antidepressants from January 1, 1995, to March 31, 2016, including their defined daily doses (DDDs), purchase dates, quantities, Anatomical Therapeutic Chemical (ATC) classification codes, and refundable purchase records. According to WHO, DDD is defined as “the assumed average maintenance dose per day for a drug used for its main indication in adults” [10]. We computed the total DDD for each medicine by multiplying the drug content of each tablet by the total purchased package size and dividing the result by the DDD of the medication as defined by WHO. For SNRI drugs, the DDD/day of drug use was related to the maximum daily dose of the same drug. The maximum dose was 120 mg/day for duloxetine and 375 mg/day for venlafaxine. We analyzed the differences in medication use in relation to retirement date. The follow-up time was limited to three years: two years before and one year after the pension. Death information was obtained from the Population Register Center, which preserves a nationwide registry of all deaths in Finland.

The study protocol was accepted by the Institutional Ethics Committee. The data synthesis from the national registries was performed with approval from the SII and the Ministry of Social Affairs and Health of Finland. This is a retrospective register study; therefore, according to Finnish law, separate patient consent was not required.

### 2.1. Statistical Analysis

The demographic data were expressed as means and standard deviations or medians and ranges. Categorical data were expressed as frequencies and percentages. Use of medication was analyzed using generalized estimation equation models with negative binomial distribution. The results were shown as means with 95% confidence intervals, and *P* values of <0.05 were considered significant. SPSS 22.0 (IBM Corporation, Armonk, New York) for Windows was used to perform all the statistical analyses.

## 3. Results

Of the 129 patients who had SCS implanted at the end of follow-up, 27 (21%) retired during the follow-up period. Of these, 48% were women (*n* = 13) (Table 1). Neuropathic pain medication was used by 17 (61%) patients, and antidepressants by 8 (29%) patients (Figure 1). Diagnosis for DP is presented in Table 2.

The mean age was 49 years (range 33–65) at the time of implantation and 48 years (range 37–60) at the time of retirement. Most of the patients suffered from both extremity and back pain (*n* = 20, 74%). The median number of previous lumbar operations before SCS implantation was two (range 1–9), and most patients underwent single-level surgery (*n* = 15, 60%). Disk herniation (*n* = 14, 52%) was the most common cause for operation. For a minority of patients (*n* = 11, 41%), a DP was granted after SCS implantation (Table 1).

### 3.1. Neuropathic Pain Medication Use during the Follow-Up

Of the 27 patients, 10 did not use any neuropathic pain medication. Pregabalin, the most-used neuropathic pain medication, was taken by nine (33%) patients six months before the DP and by seven (26%) patients 12 months after the DP. The second most-used drug was amitriptyline, which was used by five (19%) patients six months before the DP and by four (15%) patients 12 months after the DP. Antidepressants were used by eight (30%) patients at some point during the follow-up period. The most commonly used antidepressant was mirtazapine, which was used by three (11%) patients. Three patients used gabapentinoids and SNRI, two used gabapentinoids and TCA, and one used gabapentinoids, TCA, and SNRI. Three patients using antidepressants also used gabapentinoids. The other five patients using antidepressants did not take any medication for neuropathic pain (Table 3).

### 3.2. Gabapentinoid Use

**Figure 2:**
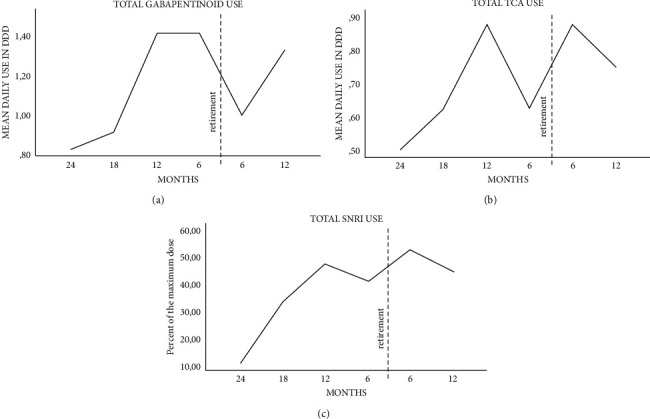
Mean daily defined doses (DDDs) of gabapentinoids (a) and tricyclic antidepressants (TCA) (b) and also percent of the maximum dose of serotonin-norepinephrine reuptake inhibitors (SNRIs) (c) 2 y before and 1 y after disability pension (DP) in persistent spinal pain syndrome patients with spinal cord stimulation at KUH. Gabapentinoid users *n* = 12, TCA users *n* = 8, and SNRI users *n* = 6. The mean DDD was calculated as the average of total purchased drugs during the specific six-month period (months: 0–6; 6–12; 12–18; 18–24 before and 0–6; 6–12 after DP). For SNRI drugs, the DDD/day of drug use was related to the maximum daily dose of the same drug. The maximum dose was 120 mg/day for duloxetine and 375 mg/day for venlafaxine.

A total of 12 patients used gabapentinoids during the follow-up. One user discontinued use after receiving a DP. One user changed the medication from pregabalin to gabapentin six months before DP. The mean gabapentinoid dose at the beginning of the follow-up was 0.8 DDD/day (95% confidence interval (95% CI): 0.4–1.6). Doses were at their highest at 6–12 months before the DP at 1.4 DDD/day (95% CI: 1.0–2.0). The increase in doses from the beginning of the follow-up to six months before the DP was also statistically significant, with a *P* value of <0.05. The mean gabapentinoid dose decreased from 1.4 DDD/day (95% CI: 1.0–2.0) six months before DP to the dose 1.0 DDD/day (95% CI: 0.7–1.5) six months after DP. At the end of follow-up, the mean dose was 1.3 DDD/day (95% CI: 0.8–2.3; Figure 2).

**Figure 3:**
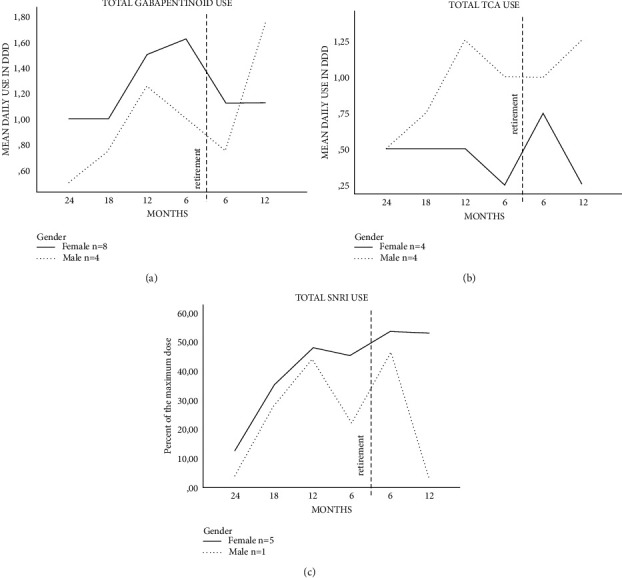
Mean daily defined doses (DDDs) of gabapentinoids (a) and tricyclic antidepressants (TCA) (b) and also percent of the maximum dose of serotonin-norepinephrine reuptake inhibitors (SNRIs) (c) 2 y before and 1 y after disability pension (DP) in persistent spinal pain syndrome patients with spinal cord stimulation at KUH. The mean DDD was calculated as the average of total purchased drugs during the specific six-month period (months: 0–6; 6–12; 12–18; 18–24 before and 0–6; 6–12 after DP). For SNRI drugs, the DDD/day of drug use was related to the maximum daily dose of the same drug. The maximum dose was 120 mg/day for duloxetine and 375 mg/day for venlafaxine.

Eight women and four men used gabapentinoids. In women, the mean use of gabapentinoids was 1.0 DDD/day (95% CI: 0.5–2.2) at the beginning of follow-up and 1.1 DDD/day (95% CI: 0.6–2.0) at the end; the dose was highest at six months before the DP at 1.6 DDD/day (95% CI: 1.1–2.3). In men, the mean use two years before the DP was 0.5 DDD/day (95% CI: 0.2–1.3) and 1.8 DDD/day (95% CI: 0.6–4.8) at the end of the follow-up (Figure 3).

**Figure 4:**
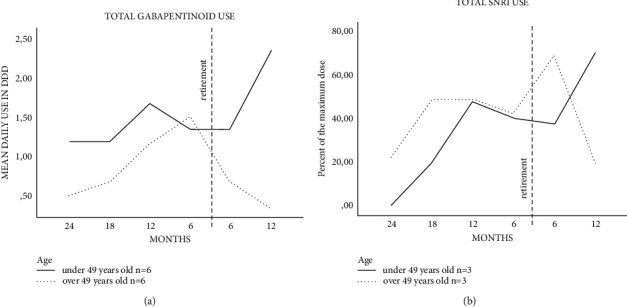
Mean daily defined doses (DDDs) of gabapentinoids (a) and percent of the maximum dose of serotonin-norepinephrine reuptake inhibitors (SNRIs) (b) 2 y before and 1 y after disability pension (DP) in persistent spinal pain syndrome patients with spinal cord stimulation at KUH. The mean DDD was calculated as the average of total purchased drugs during the specific six-month period (months: 0–6; 6–12; 12–18; 18–24 before and 0–6; 6–12 after DP). For SNRI drugs, the DDD/day of drug use was related to the maximum daily dose of the same drug. The maximum dose was 120 mg/day for duloxetine and 375 mg/day for venlafaxine.

Half (*n* = 6) of the gabapentin users were under 49 years old. In those under 49 years old, the median dose two years before the DP was 1.2 DDD/day (95% CI: 0.6–2.4) and one year after the DP, it was 2.3 DDD/day (95% CI: 1.7–3.2). The corresponding figures for users over 49 years old were 0.5 DDD/day (95% CI: 0.1–1.7) and 0.3 DDD/day (95% CI: 0.6–2.0). For users over 49 years old, the mean dose peaked at six months before the DP at 1.5 DDD/day (95% CI: 0.9–2.5), with a *P* value of <0.05 compared to the beginning of the follow-up (Figure 4).

### 3.3. TCA Use

TCAs were used by eight patients during the follow-up. After the DP, use was discontinued by two patients. At the beginning of the follow-up, the mean TCA use was 0.5 DDD/day (95% CI: 0.2–1.3), and it reached 0.8 DDD/day (95% CI: 0.3–1.8) at the end of the follow-up. The mean TCA use was at its highest within 12 months before and six months after the DP at 0.9 DDD/day (95% CI: 0.4–1.8). All TCA users were under 49 years old (Figure 2).

Of TCA users, four were men and four were women. In men, the mean dose was 0.5 DDD/day (95% CI: 0.1–2.7) at the beginning of the follow-up and 1.3 DDD/day (95% CI: 0.5–2.9) at the end of the follow-up, with a *P* value of <0.05. In women, the corresponding figures were 0.5 DDD/day (95% CI: 0.2–1.3) and 0.3 DDD/day (95% CI: 0.05–1.4; Figure 3).

### 3.4. SNRI Use

A total of six patients used SNRI during the follow-up. The mean SNRI dose at the beginning of the follow-up was 11% of the maximum dose (95% CI: 2.1–58). The dose gradually increased thereafter, reaching its highest at the end of the follow-up, six months after retirement, at a mean of 52% of the maximum dose (95% CI: 33–85), with a *P* value of <0.05 (Figure 2).

During the follow-up, SNRI was used in five women and one man. In women, the mean dose was at its lowest two years before the DP at 12.4% of the maximum dose (95% CI: 2.1–71.6) and at its highest six months after the DP at 54% of the maximum dose (95% CI: 31.1–93.8), with a *P* value of 0.05 (Figure 3).

Half (*n* = 3) of SNRI users were under 49 years old. In patients under 49 years old, the mean dose was 19.3% of the maximum dose (95% CI: 4.4–85.0) 18 months before the DP and 70.3% of the maximum dose (95% CI: 44.1–112.2) 12 months after the DP, with a *P* value of 0.05. In patients over 49 years old, the mean dose was at its highest at six months after the DP at 68.7% of the maximum dose (95% CI: 44.8–105.1) and at its lowest 12 months after the DP at 19% of the maximum dose (95% CI: 4.3–83.1), with a *P* value of 0.05 (Figure 4).

## 4. Discussion

Of the 230 patients trialed for SCS, permanent IPG hardware was installed in 179 (78%), and 129 (72%) used the hardware at the end of the follow-up. During data collection, 28 out of 129 (21.7%) SCS permanent patients had a DP, and 27 had sufficient follow-up time (two years before and one year after retirement). In this study, patients constituted consecutive PSPS patients with SCS in a single publicly funded tertiary center, who are beneficiaries of the public pension system covering all residents in Finland.

This study analyzed the use of neuropathic pain medication from two years before to one year after receiving a DP in 27 PSPS patients with an implanted SCS. PSPS is a complex chronic pain problem in which psychosocial factors play a major role. Medication is one part of the treatment of chronic pain and is also a first-line treatment. We found that most of the patients (61%) used neuropathic pain medications during the follow-up, and the use appeared to be regular, with relatively few medication initiations and discontinuations.

Antidepressants were used by a third of the patients—or by 44% if SNRI use is counted. The majority (70%, *n* = 19) of the patients retired because of dorsopathies. The dose of gabapentinoids started to increase before the DP; after the DP, the doses started to increase again after the decrease, but the doses remained at a lower level after the DP. This is explained by the decrease in doses for women and patients over 49 years old. The use of gabapentinoids began to decline after the DP, but the dose returned to almost pretreatment levels as early as 12 months after the DP. A probable explanation is that for some people, getting a retirement decision is a relief or reduces stress or possibly even physical burden. This would be indirectly reflected in a decrease in the dosage of neuropathic pain medications. On the other hand, the change is often short term and over time the drug dose returns to the previous level. The mean dose of TCA increased after the DP, and the increase in SNRI was steady toward the end of the follow-up.

Neuropathic pain is particularly common in women and in patients over 50 years old [11]. In our data, gabapentinoid use increased in patients under 49 years old throughout the follow-up and was more pronounced after the DP. By contrast, in patients over 49 years old, the dose decreased after the DP. Women used more gabapentinoids than men, and their dose decreased after the DP, while in men, the mean dose increased after the DP. Women also used more SNRI drugs, while during the follow-up, the drug was used by only one man. Women are generally more open about mood issues, and this may be a possible factor why SNRI drugs are used more by them. In this study, TCA was used only in patients under 49 years old. Men used TCA at a higher dose compared to women, which may be due to a difference between the sexes, but this issue is still controversial [12]. Lower medication uses in patients over 50 years old may be explained by comorbidities and drug interactions.

The strengths of this study are its homogenous cohort of consecutive PSPS patients with a long follow-up period. Patient care is publicly funded, and the Finnish pension system covers all residents, both of which reduce the bias of the study. Data on pensions and medication use were obtained from the national prospective register and patients' follow-up without dropouts. The analyses were based on medical records, national register data of DPs, and the national registry of reimbursed medicines for all purchases of prescribed gabapentinoids and antidepressants. The patients' use of medications and their retirement are in themselves objective quantities of a patient's functional capacity.

This is a retrospective study with apparent limitations. Structured questionnaires were not used for this study population, and data on the social situation of patients were missing. We also have no information on rehabilitative interventions or other therapies for chronic pain before the SCS. This was a long-term follow-up study, and during the time in which it was executed, the understanding of chronic pain underwent changes. The criteria for the implantation of permanent SCS devices have also changed, and SCS devices and surgical techniques have progressed. Unfortunately, the programming data of the SCS equipment were not available in this patient group, and thus it remains unclear whether the patients received optimal SCS treatment. Regarding further research, it would be important to evaluate how SCS programming affects patient outcomes and medication use. Also, it would be interesting to repeat the study in a genetically and socially less homogenous population than that exists in Finland.

## 5. Conclusions

Based on neuropathic pain medication and antidepressant use, the results showed that neuropathic pain continued after DPs—that is, pensioners continued to experience inconvenient chronic pain. Resources for patient care are therefore needed after a DP. However, the dose increase of gabapentinoids was reduced by a DP, with the dose higher just before retirement compared to the end of the follow-up in patients over 49 years old and in women.

## Figures and Tables

**Table 1:** Demographics of 27 persistent spinal pain syndrome patients with spinal cord stimulation and disability pension collected in the Kuopio University Hospital in the period of 1996-2014.

		SCS in use at the end of follow-up *n* = 27
Gender	Female	13 (48%)

Age on pension date (mean ± SD)		48 ± 6.5

Location of pain	Extremity	7 (26%)
	Extremity and back	20 (74%)

Number of previous operations before implantation (median/range)		2/1–9

Level of operation (*n* = 25)	L4-5 and above	8 (32%)
	L5-S1	7 (28%)
	Multiple levels	10 (40%)

Reason for operation	Disc herniation	14 (52%)
	Stenosis	4 (15%)
	Disc herniation and stenosis	9 (33%)

Type of electrode^a^	Symmix/Resume 1 × 4/Vectris	21 (78%)
	Specify 5-6-5/2 × 4	6 (22%)

Electrode location	T8-9	8 (30%)
	T9-10	13 (48%)
	T10–11	4 (15%)
	T11–12	2 (7%)

Disability pension granted after implantation		11 (41%)

SCS = spinal cord stimulation; ^a^all electrodes manufactured by Medtronic, Minneapolis, MN, USA.

**Table 2:** Disability pension diagnosis of 26 persistent spinal pain syndrome patients with spinal cord stimulation during a follow-up of 3 years based on data from the Social Insurance Institution of Finland.

Primary diagnosis (collateral diagnosis)	SCS in use at the end of follow-up *n* = 26
*F00–F99 mental and behavioural disorders*	
F30–39 mood (affective) disorders	3 (1)
F40–48 neurotic, stress-related, and somatoform disorders	(1)
F60–69 disorders of adult personality and behaviour	(1)

*G00–99 diseases of the nervous system*	
G50-G59 nerve, nerve root, and plexus disorders	(1)

*M00–99 diseases of the musculoskeletal system and connective tissue*	
M00–25 arthropathies	1
M40–54 dorsopathies	19 (5)
M60–79 soft tissue disorders	1
M95–99 other disorders of the musculoskeletal system and connective tissue	2

SCS = spinal cord stimulation.

**Table 3:** Neuropathic pain medication and antidepressant use among 27 patients with spinal cord stimulation for persistent spinal pain syndrome 6 months before and 12 months after entering disability pension.

	Users 6 months before disability pension		Users 12 months after the disability pension	
	Not in use (*n*)	In use (*n*)	Not in use (*n*)	In use (*n*)
*Gabapentinoids, SNRI, TCA*				
Gabapentin (N02BF01)	23	4	23	4
Pregabalin (N02BF02)	18	9	20	7
Venlafaxine (N06AX16)	24	3	23	4
Duloxetine (N06AX21)	25	2	26	1
Amitriptyline (N06AA09)	22	5	23	4
Nortriptyline (N06AA10)	26	1	26	1

*Antidepressants*				
Mianserin (N06AX03)	26	1	26	1
Mirtazapine (N06AX11)	25	2	25	2
Milnacipran (N06AX17)	26	1	26	1
Reboxetine (N06AX18)	27	0	27	0
Fluoxetine (N06AB03)	27	0	27	0
Citalopram (N06AB04)	26	1	26	1
Paroxetine (N06AB05)	27	0	26	1
Fluvoxamine (N06AB08)	26	1	26	1
Escitalopram (N06AB10)	27	0	27	0

## Data Availability

Background information supporting the results is available from the corresponding author if necessary.
